# The transcriptional regulatory network of hormones and genes under salt stress in tomato plants (*Solanum lycopersicum* L.)

**DOI:** 10.3389/fpls.2023.1115593

**Published:** 2023-02-06

**Authors:** Baike Wang, Juan Wang, Tao Yang, Jinxin Wang, Qi Dai, Fulin Zhang, Rui Xi, Qinghui Yu, Ning Li

**Affiliations:** ^1^ Key Laboratory of Genome Research and Genetic Improvement of Xinjiang Characteristic Fruits and Vegetables, Institute of Horticultural Crops, Xinjiang Academy of Agricultural Sciences, Urumqi, Xinjiang, China; ^2^ Research Institute of Soil, Fertilizer and Agricultural Water Conservation, Xinjiang Academy of Agricultural Sciences, Urumqi, Xinjiang, China; ^3^ College of Horticulture, Xinjiang Agricultural University, Urumqi, Xinjiang, China

**Keywords:** tomato, salt stress, hormone, RNA-seq, WGCNA

## Abstract

Salt stress has become one of the main limiting factors affecting the normal growth and development of tomatoes as well as fruit quality and yields. To further reveal the regulatory relationships between tomato hormones under salt stress, the interaction between hormones and TF and the genome-wide gene interaction network were analyzed and constructed. After salt treatment, the levels of ABA, SA, and JA were significantly increased, the levels of GA were decreased, and IAA and tZ showed a trend of first increasing and then decreasing. The expression patterns of hormone biosynthesis and signal transduction related genes were analyzed based on RNA-seq analysis, the co-expression network of hormones and genome-wide co-expression networks were constructed using weighted gene co-expression network analysis (WGCNA). The expression patterns of specific transcription factors under salt stress were also systematically analyzed and identified 20 hormone-related candidate genes associated with salt stress. In conclusion, we first revealed the relationship between hormones and genes in tomatoes under salt stress based on hormone and transcriptome expression profiles and constructed a gene regulatory network. A transcriptional regulation model of tomato consisted of six types of hormones was also proposed. Our study provided valuable insights into the molecular mechanisms regulating salt tolerance in tomatoes.

## 1 Introduction

Tomato (*Solanum lycopersicum* L.) is a perennial herbaceous plant of family Solanaceae copiously cultivated over many places worldwide and occupies an important position in the agricultural economy and food security production with high economic value (Sato et al., 2012). Excessive salinity in the soil had become one of the main limiting factors for normal tomato growth and development (Cuartero et al., 2006). Hypersaline environments could affect or delay the onset of seed germination in tomatoes, but it was usually limited by the level of salinity (Cuartero et al., 2006). With the increasing development of tomato facility cultivation, the problems caused by soil salinization had become increasingly prominent. It could significantly affect nutrient and water uptake efficiency in tomatoes and thus affected the normal growth and physio-logical metabolism of tomato plants, resulting in slower growth and lower yields (Deinlein et al., 2014).

The dynamic changes in hormone had a substantial impact on the normal growth and development of plants under salt stress (Yu et al., 2020). The accumulation of ABA could regulate stomatal closure, ion homeostasis, gene expression, and metabolic changes in response to salt stress, and hence reduce the influences of salt-alkali stress (Raghavendra et al., 2010). When plants were ex-posed to salt stress, the accumulated IAA and CK in root tips could confer augmented re-sistance (Yu et al., 2020). Previous studies had found that the expression of the IAA-related genes *SAUR32*, *SAUR36*, and *ARF5* and the cell division-related gene IPT5 could be significantly induced in the roots of apple rootstocks under salt stress. These genes could improve the salt tolerance by increasing the IAA and CK content in the apple rootstocks (Quint and Gray, 2006). JA could inhibit plant growth and root elongation by activating antioxidant enzymes, delay plant flowering and improve plant viability under salt stress (Ali and Baek, 2020). Additionally, SA and GA were also crucial in salt stress (Khan et al., 2019).

High-throughput RNA sequencing (RNA-seq) had become one of widespread methods for measuring transcriptional expression profiles of genes (Natukunda et al., 2022). Based on the temporal gene expression analysis, Kuang et al. had generated the gene regulatory network of fruit ripening in banana (Kuang et al., 2021). Garg et al. had constructed several regulatory networks related to chickpea development (Garg et al., 2017). Weighted gene co-expression network analysis (WGCNA) could also offer foundation for the mining of potential hub genes using hierarchical clustering (Ren et al., 2022). These findings demonstrated that the identification of regulatory networks might be effective for precisely understanding transcriptional regulatory mechanisms by using transcriptome analysis.

Wild tomatoes were originated in South America and exhibited greater salt tolerance than cultivated tomatoes under hypersaline environments. Cultivated tomatoes had gradually lost their capacity to adapt to high salt stress during the domestication process (Sato et al., 2012). At present, preliminary studies on mechanisms of salt tolerance in tomato had been reported (Yang et al., 2022), but the hormone-related regulatory network of tomato plants under salt stress remained unclear. Further studies on the regulatory networks of hormone metabolism and signal transduction might be important to precisely elucidate the salt stress mechanism of tomato plants. To analyze the temporal relationship and coordinated interactions between the metabolic regulatory networks of tomato hormones under salt stress, the hormone analysis and transcriptome profiling analysis were carried out, and the photosynthetic, physiological, and biochemical changes of tomato plants in response to abiotic stress were also studied systemically. We investigated the interaction between hormones and hormone signalling pathways and constructed a transcriptional regulation model for tomato genes under salt stress to laid the groundwork for further analyzing molecular mechanisms of salt tolerance.

## 2 Result

### 2.1 Changes in physiological and photosynthetic indicators

**Figure 1:**
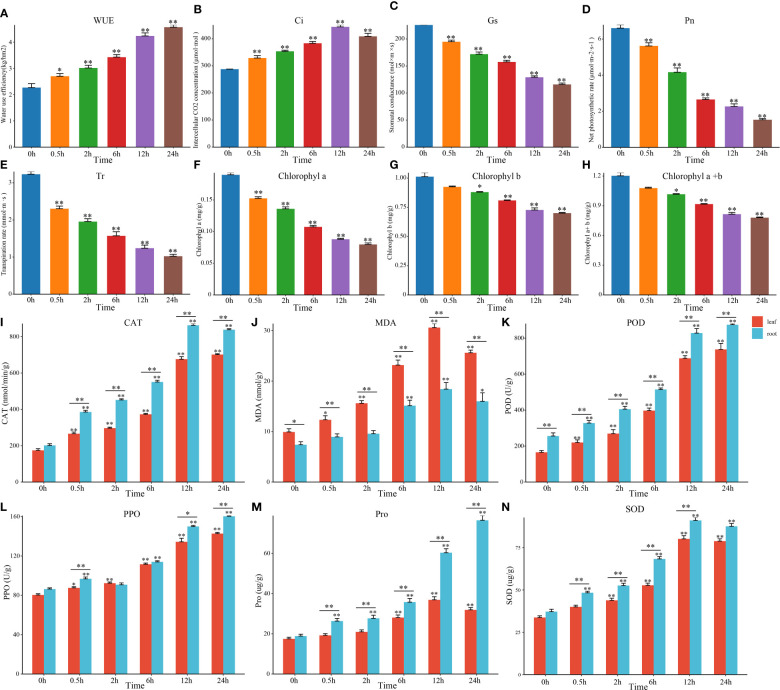
Changes in tomato photosynthesis and physiology under salt stress. **(A–H)** Changes in photosyn-thesis and the chlorophyll content in tomato leaves at DTP under NaCl treatment. **(I–N)** Changes in tomato physiological indices at DTP under salt stress. (*P-value < 0.05; **P-value < 0.01).

In recent years, salt stress had been confirmed to affect the morphological structure of plant photosynthetic organs, photosynthesis processes, antioxidant systems, and osmotic adjustment substance contents in a variety of plants (Ma et al., 2017). For our initial evaluation, physiological and photosynthetic markers were utilized to discover the reduction of Gs, Pn, Tr, and chlorophyll content and significant increasing concentration of the WUE and Ci in tomato leaves under salt stress (**Figures 1A–H**). The contents of CAT, MDA, POD, PPO, Pro and SOD in tomato leaves and roots also increased significantly (**Figures 1I–N**). But the changes in roots were significantly greater than those in leaves, which could be relevant to where the location of salt stress occurring. In summary, we evaluated the photosynthetic and physiological indicators of tomato seedlings and discovered several situations under salt stress. For instance, the photosynthetic system of tomato leaves was devastated and resulting in the inhibited photosynthetic efficiency. At the same time, the stomatal opening was inhibited and the content of protective enzymes was also increased. To further analyze its molecular mechanism and the interaction between hormones, we determined the hormone content and performed the transcriptome assays at different time points (DTP) under salt treatment.

### 2.2 ABA content and transcriptional changes

**Figure 2:**
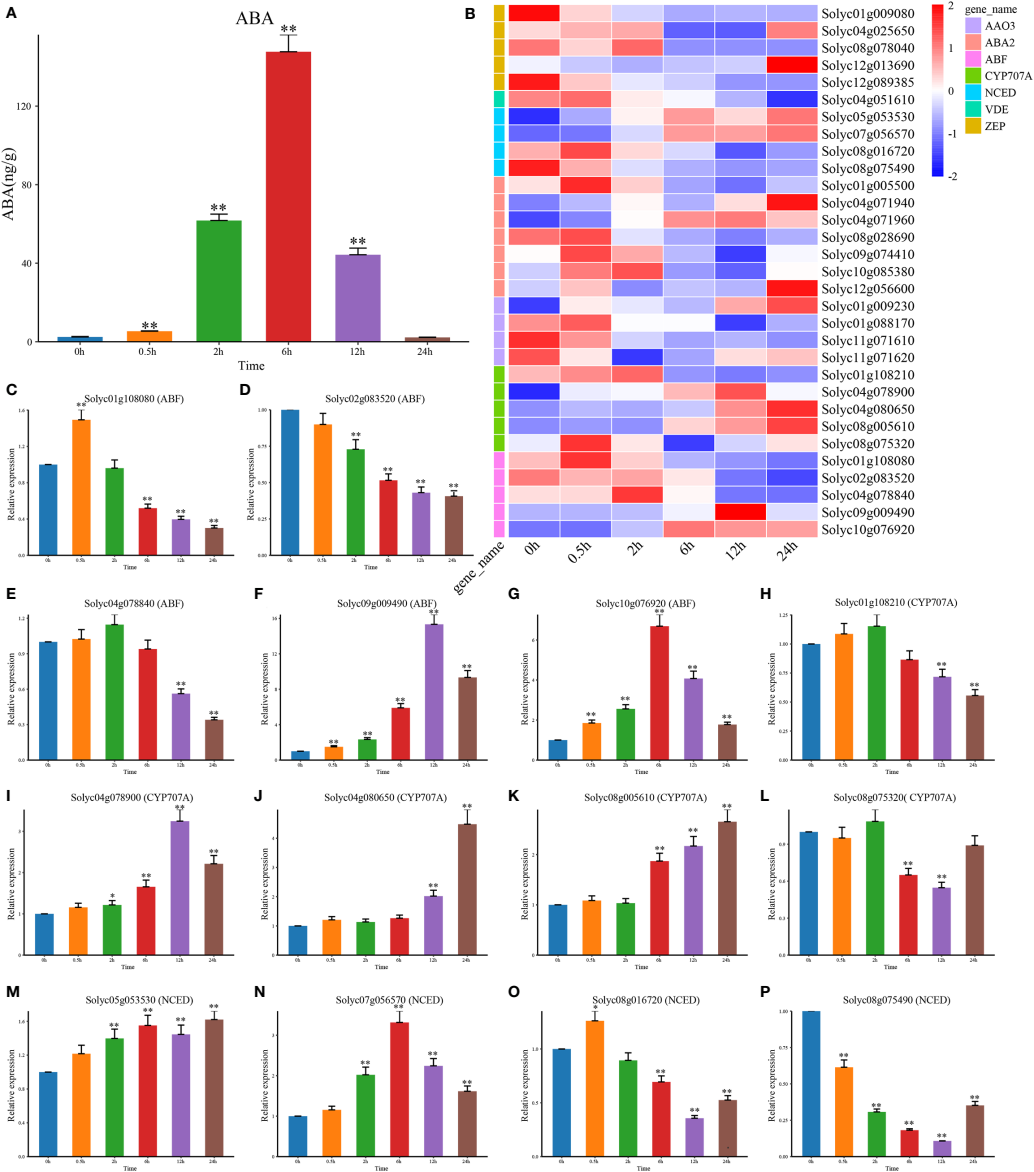
The content of hormone ABA and the NaCl treatment’s effects on the expression patterns of the genes involved in ABA biosynthesis and signal transduction. **(A)** The level of the hormone ABA at DTP under salt stress **(B)** Following the NaCl treatment, the expression profiles of DEG involved in ABA biosynthesis and signal transduction at DTP **(C–P)** The expression patterns of CYP707A, NCED, and ABF at DTP under salt stress. (*P-value < 0.05; **P-value < 0.01).

The response to salt stress in plants was mainly governed by the changes in endogenous ABA levels (Quint and Gray, 2006; Khan et al., 2019). Accordingly, we first determined the content of ABA and found that it was greatly rose and peaked at 6 h (**Figure 2A**). By RNA-seq expression analysis, we examined the expression patterns of DEGs which involved in ABA biosynthesis pathways (**Figure 2B**). Among them, several *CYP707A* genes were highly expressed in a short period of 1 h and then gradually decreased, but the expression levels of *NCED* and *AAO3* were elevated after 3 h under salt treatment. This suggested that the expression of *CYP707As* might contribute to the transient accumulation of endogenous ABA, while *NCED* and *AAO3* might be involved in the later stages of ABA accumulation under salt stress. There-fore, we hypothesize that the interaction between *NCED3*, *AAO3*, and *CYP707As* could control the ABA levels in tomato plants. By analyzing the genes associated with ABA signal transduction pathways, we found that only the positive regulators ABF were differentially expressed and upregulated at DTP under salt stress (**Figure 2B**). These results suggested that the content of endogenous ABA in tomato plants might be mainly regulated by the expression of the *AAO3*, *ABA2*, *CYP707A*, *NCED*, *VDE*, and *ZEP* genes, while the increasing ABA content might induce ABF to regulate some physiological processes. We then selected three core genes of ABA biosynthesis and signal transduction pathways (*CYP707A*, *NCED*, and *ABF*) to perform qRT-PCR and found that CYP707A was significantly upregulated, except for *Solyc01g108210* and *Solyc08g075320* (**Figures 2H–L**). Apart from *Solyc08g016720* and *Solyc08g075490*, the other *NCED* genes were also significantly up-regulated (**Figures 2M–P**). However, only *Solyc09g009490* and *Solyc10g076920* were significantly upregulated among the *ABF* genes which involved in the ABA signal transduction pathway (**Figures 2C–G**). These results indicated that the genes related to the ABA pathways had both positive and negative regulatory roles and also exhibited complex signaling crosstalk to control the response to salt stress in tomato plants under salt stress.

### 2.3 Effects of salt stress on JA and SA biosynthesis and signal transduction

**Figure 3:**
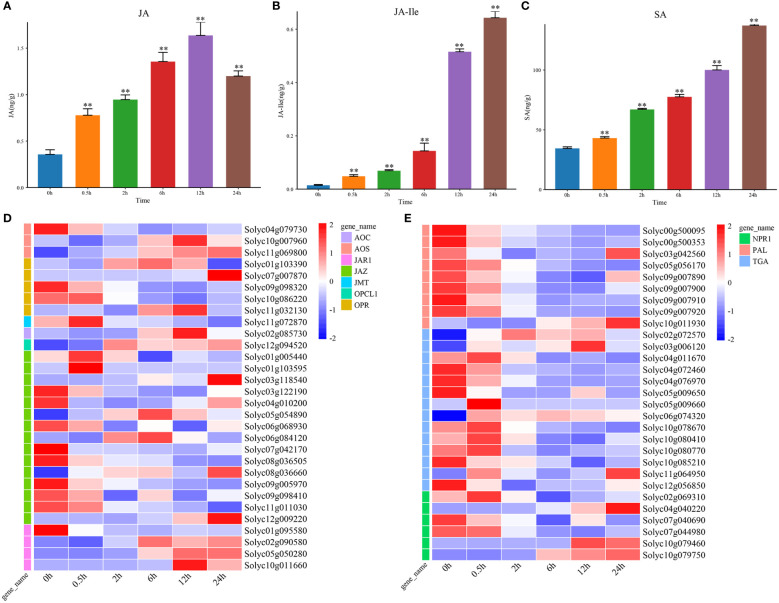
The contents of hormone JA and SA, and expression profiles of signal transduction genes under salt stress. **(A)** The content of hormone JA at DTP under salt stress **(B)** The level of hormone JA-Ile at DTP under salt stress **(C)** The content of hormone SA at DTP under salt stress **(D)** The ex-pression profiles of DEGs involved in JA biosynthesis and signal transduction at DTP under salt stress **(E)** The expression profiles of DEGs involved in SA biosynthesis and signal transduction at DTP under salt stress (**P-value < 0.01).

As the positive regulators of salt stress, JA and SA also played important roles in response to salt stress (Khan et al., 2019; Ali and Baek, 2020). We found that the levels of JA and JA-Ile was significantly increased at 0.5 h under salt stress (**Figures 3A, B**). The genes encoding the rate-limiting enzymes that were involved in JA biosynthesis, like *AOC*, *AOS*, *JMT*, *OPCL1*, and *OPR* were upregulated at DTP under salt stress, and these changes were more obvious along with the prolonged treatment time. In addition, only *Solyc04g079730*, *Solyc09g098320*, *Solyc10g086220*, and *Solyc01g095580* were downregulated. These results illustrated that the levels of JA and JA-lle might be negatively regulated by the four genes (**Figure 3D**). However, as one of the regulators of signal transduction pathways, *JAR1* reached peak expression at 12 h under salt stress, which was consistent with the change in JA content. The JA signal transduction related gene *JAZ* was down-regulated. This result showed that the increase in MeJA content could induce the expression of *JAR1* rapidly and then promote the synthesis of JA-Ile. The expression of JAZ was also regulated to improve the salt tolerance of the tomato plants (**Figure 3D**). From these, it is reasonable to hypothesize the JA homeostasis in tomatoes might be related to the transcriptional regulation of ABA biosynthesis and signal transduction related genes and that elevated ABA levels could upregulate the JA levels. It was also possible that the increase in ABA content *in vivo* could induce the expression of JA biosynthesis related genes and thereby regulate the content of endogenous JA and MeJA in tomatoes.

In plants, the changes in SA content could be regulated by signaling molecules *in vivo* and the external environments. The salt stress could induce marked changes in SA content (Khan et al., 2019). By the determination of SA content, we found that salt stress could result in the increasing SA content (**Figure 3C**). Two SA biosynthesis related genes (*Solyc10g011930* and *Solyc03g042560*) were also found to be highly expressed and this showed that the increasing SA content was related with the inducible expression of them (**Figure 3E**). Five SA signal transduction related genes (*Solyc02g069310*, *Solyc04g040220*, *Solyc10g079460*, *Solyc10g079750* and *NPR1*) were upregulated, while TGA transcription factors were generally showed a decreasing trend (**Figure 3E**). These comprehensive results suggested that salt stress treatment could promote the continuous increase in SA levels *in vivo* by inhibiting the expression of SA signal transduction related genes.

### 2.4 Effects of salt stress on IAA, GA, and tZ biosynthesis and signal transduction

**Figure 4:**
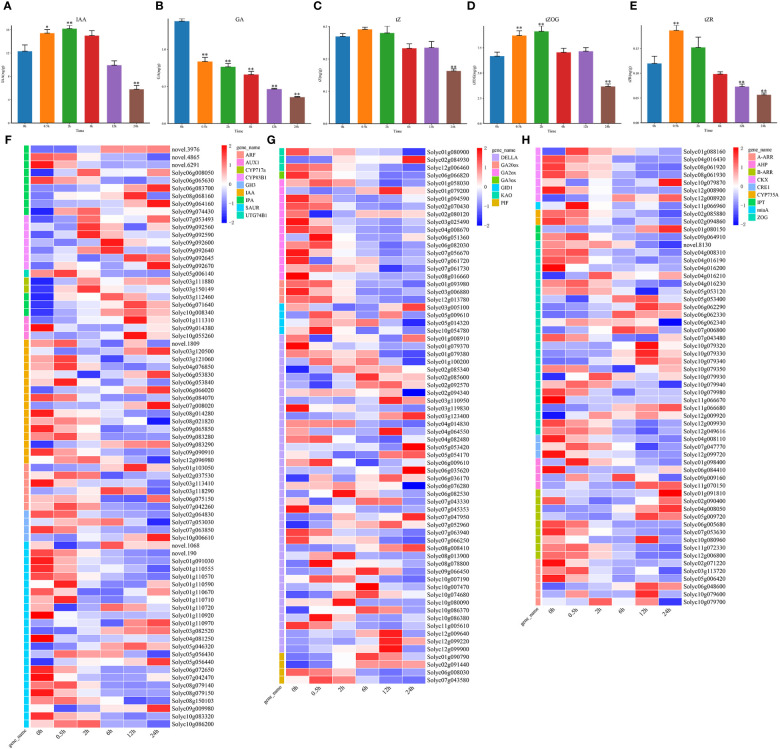
The changes in hormone IAA, GA, and tZ contents and expression patterns of genes involved in hormone biosynthesis and signal transduction under salt stress. **(A)** The hormone IAA levels at DTP under salt stress **(B)** The level of hormone GA at DTP under salt stress **(C–E)** The tZ, tZR, and tZOG hormone contents at DTP under salt stress **(F)** Expression patterns of DEGs involved in signal transduction and IAA production at DTP under salt stress **(G)** The expression profiles of DEGs involved in GA biosynthesis and signal transduction at DTP under salt stress **(H)** the expression profiles of DEGs involved in tZ biosynthesis and signal transduction at DTP under salt stress. (*P-value < 0.05; **P-value < 0.01).

The changes in IAA content during salt stress was complex (**Figure 4A**). Except for the 4 IPA genes (*novel.3976*, *novel.4865*, *novel.6291*, and *Solyc06g065630*), the other IAA biosynthesis related genes, like *CYP717a*, *CYP83B1*, *IPA*, and *UTG74B1* were consistent with the trend of the IAA content. The genes involved in the IAA signal transduction pathway were all downregulated (**Figure 4F**). These indicated that the increase in IAA content might be due to the downregulated expression of signal transduction related genes.

The GA level showed a continuous decreasing trend during salt stress (**Figure 4B**). Except for *Solyc02g084930*, *Solyc01g079200*, and *Solyc06g051360*, the GA biosynthesis related genes, *GA20ox*, *GA2ox*, *GA3ox*, and *KAO* were all downregulated at 0.5 h, which coherently with the trend of GA content variation (**Figures 4B, G**). We focused on the GRAS family protein DELLA, a negative regulator of GA signaling. The complex expression patterns of DELLA genes contained both upregulation and downregulation (**Figure 4G**). The expression of the GA signal transduction related genes *PIF* and *GID1* were also upregulated, which indicated that the reduction in endogenous GA levels in the tomato plants under salt stress was mainly regulated by the GA biosynthesis related genes. The complexity of the GA signal transduction related gene expression might be related to the signaling crosstalk between hormones and this process requires further elucidation.

Under salt stress, tZ (trans-Zeatin Riboside) content was only considerably decreased at 24 h (**Figure 4C**), trans-ZEATIN-O-GLUCOSIDE (tZOG) content was significantly increased at 0.5 h and significantly decreased at 24 h (**Figure 4D**). Moreover, the trans-Zeatin Riboside (tZR) trend was seen to closely resemble the tZOG trend (**Figure 4E**). We analyzed the expression patterns of the tZ biosynthesis and signal transduction related genes (**Figure 4H**). The biosynthetic genes *AHP*, *CKX*, *CYP735A*, *IPT*, *miaA*, and *ZOG* were downregulated, and only some *ZOG* genes were upregulated (**Figure 4H**). In addition to 8 genes (*Solyc09g009160*, *Solyc11g070150*, *Solyc01g091810*, *Solyc04g008050*, *Solyc05g009720*, *Solyc06g048600*, *Solyc10g079600*, and *Solyc10g079700*), *CRE1*, *AHP*, *B-ARR*, and *A-ARR* were all downregulated. Although the changes in endogenous tZ content were not significant, the genes related to the biosynthesis and signal transduction were significantly upregulated or downregulated, these indicated that the signaling crosstalks between hormones were very complex, and further exploration and research is needed.

### 2.5 Global transcriptome changes under salt stress

**Figure 5:**
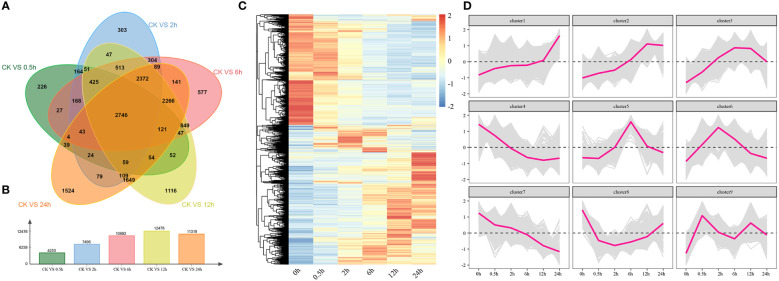
The results of differentially expressed analyses and cluster analyses. **(A)** The amount of shared and different genes that were expressed at DTP under salt stress **(B)** The number of DEGs at DTP under salt stress **(C)** The heatmap showed the distinctive expression patterns of DEGs at DTP under salt stress **(D)** The line graph showed the expression changing trends of genes that were contained in clusters.

The correlation and PCA analysis of transcriptome data were performed to demonstrate the reliability of the transcriptome data (**Figure S1**). Through analyzing the differentially expressed genes between 0 h and other time points, we found 4520, 7496, 10692, 12476, and 11319 DEGs at 0.5 h, 2 h, 6 h, 12 h, and 24 h, respectively (**Figures 5A, B**). There were also 2746 DEGs found to be differentially expressed at all time points under salt stress and the period-specific DEGs increased with salt stress duration (**Figure 5A**). Through the hierarchical clustering, we also found 9 statistically significant clusters and generated several expression profiles of pulses (**Figures 5C, D**). Among them, Cluster 1, Cluster 2, and Cluster 3 showed the overall upregulated trends, while Cluster 4 and Cluster 7 showed the overall downregulated trends at DTP. These results illustrated that the various expression patterns of these genes might coordinate the tomato tolerance to salt stress.

### 2.6 Construction of the hormone-related co-expression network and TF analysis

**Figure 6:**
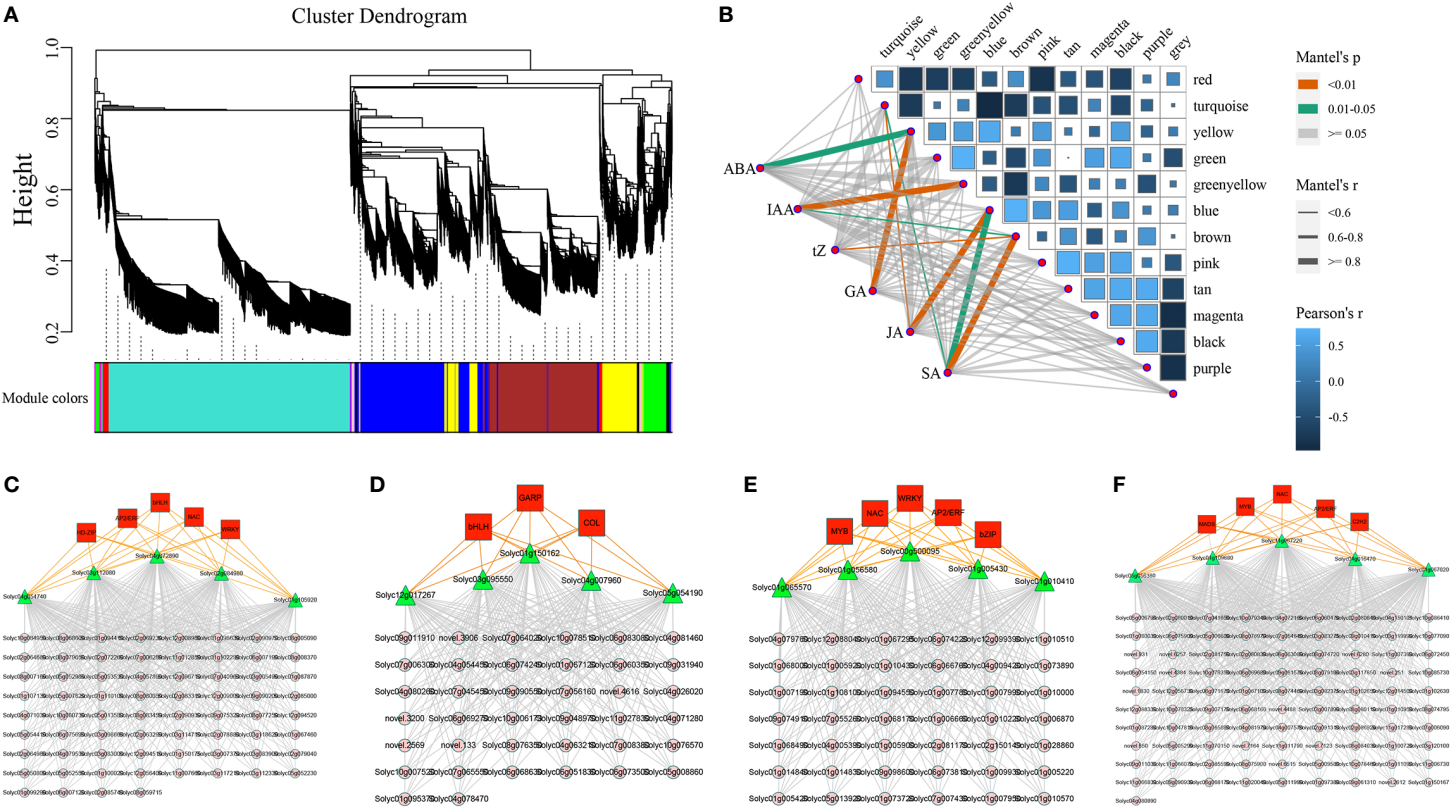
The correlations between different gene modules and hormones, co-expression networks, and the expression profiles of major transcription factors under salt stress. **(A)** The division of different gene modules **(B)** The heatmap illustrated the relationships between modules and hormones **(C–F)** The gene co-expression networks consisted of transcription factors, target genes, and hub genes for the yellow, green-yellow, blue, and brown modules.

To reveal the function of the regulatory networks rather than that of individual genes, we constructed a salt stress related co-expression network for the tomato plants by using an unsupervised network analysis approach (WGCNA), which contained the genes with similar expression patterns. The results of WGCNA of DEGs revealed 15 modules of co-expressed genes (**Figure 6A** and **S2**). We went on to further calculate the correlations between modules and hormone content, in which yellow was highly correlated with ABA and GA, blue was highly correlated with both JA and SA, while green-yellow and brown were highly correlated with IAA and SA, respectively (**Figure 6B**). The yellow module mainly consisted of 5 TF of five different types (AP2/ERF, bHLH, HD-ZIP, NAC, and WRKY), 119 target genes, and 5 hub genes (**Figure 6C**). The green-yellow module mainly consisted of 3 TFs of five different types (bHLH, COL, and GARP), 126 target genes, and 5 hub genes (**Figure 6D**). The blue module mainly consisted of 5 TFs of five different types (AP2/ERF, bZIP, MYB, NAC, and WRKY), 119 target genes, and 5 hub genes (**Figure 6E**). The brown module mainly consisted of 5 TFs of five different types (AP2/ERF, C2H2, MADS, MYB, and NAC), 126 target genes, and 5 hub genes (**Figure 6F**). Based on qRT-PCR results, we also revealed the expression patterns of the 20 hub genes in the modules under salt stress (**Figure S3**). To further study the expression patterns of transcription factors, we used RNA-seq to analyze the expression patterns of 11 TF genes which could be classified into 5 different types under salt stress (**Figure S4**). Altogether, we found that most genes of these 11 different transcription factor types were upregulated at 2 h under salt stress, and the expression patterns were closely matched with each other. We also identified the core genes of these four modules, which could act as potential candidates for futher mining salt tolerance related genes in tomatoes.

### 2.7 Effects of salt stress on transcription factor expression and the construction of genome-wide co-expression networks

**Figure 7:**
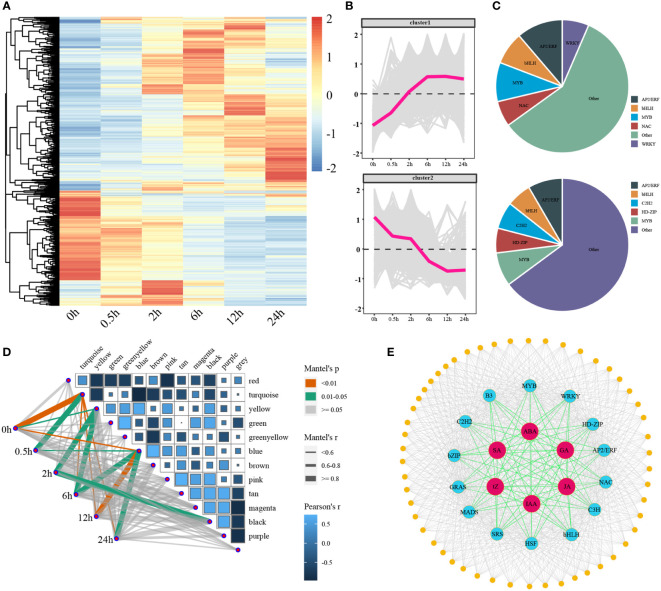
The expression patterns of differentially expressed transcription factors under salt stress and the correlations between the modules and stages, as well as the genome-wide co-expression network. **(A, B)** The heatmaps and line charts of the differentially expressed transcription factors **(C)** The ratio of core up- and down-regulated transcription factors **(D)** A heatmap of the relationships between modules and time points, the width of lines reflected the strength of correlations **(E)** A genome-wide co-expression network of hormones, core transcription factors, and target genes.

There were 1257 transcription factors were differentially expressed and the expression profiles of them clearly distinguished the two groups: up-regulated and down-regulated (**Figures 7A, B**). Five transcription factor families were found to be up-regulated, including AP2/ERF, NAC, MYB, bHLH, and WRKY (**Figure 7B**). Simultaneously, five transcription factor families were down-regulated, including AP2/ERF, HD-ZIP, acetylene, bHLH, and MYB (**Figure 7C**). As TFs could mediate repression of targets, there should have a high correlation between TFs and target genes. We determined the relevance between the 13 modules which previously examined by WGCNA and treatment time points (**Figure 7D**). We selected the yellow, green, blue, and black modules which were significantly highly correlated with DTP under salt stress. A genome-wide co-expression network of the hormone-related genes, transcription factors, and co-regulated genes was constructed in tomato. We identified a total of 14 classes of TFs (AP2/ERF, GRAS, C2H2, MYB, C3H, bHLH, bZIP, HD-ZIP, HSF, MADS, B3, NAC, SRS, and WRKY) and 52 CO binding genes (**Figure 7E**). We also examined the expression patterns of 14 TFs extensively (**Figure S5**). In conclusion, our study provided a foundation for further research of the molecular mechanisms underlying tomato salt tolerance by revealing the regulatory network of hormone signaling pathways and gene-regulatory interactions firstly in the processes of tomatoes subjected to salt stress.

## 3 Discussion

Excessive soil salinity was one of the primary inhibitors that restricted typical plant development and growth and plants had evolved special adaptive mechanisms to cope with salt stress (Sun et al., 2022). With the continuous increase of salt content in saline-alkali land, the increased osmolarity of soil solution could restrict the Rate of root water uptake and lead to the phenomenon of physiological drought in plants (Flowers and Colmer, 2015). Chen found that the decrease in plant osmotic potential rate was higher than that of the plant moisture content potential with increasing salt stress time (Hu et al., 2022). Earlier studies indicated that MDA could act as the main osmotic regulator and important maker of oxidant stress in plants under salt stress. Moreover, the increase in proline content could also be beneficial to improve the water retention capacity of cells under salt stress (Xu et al., 2021; Zhang et al., 2022). When tomatoes were subjected to salt stress, large amounts of reactive oxygen species (ROS) was induced to generate (Apel and Hirt, 2004; Waszczak et al., 2018), and the activity of SOD, POD, CAT and other ROS scavengers was accordingly increased to remove the excess ROS to maintain the redox equilibrium in the plant (**Figure 1**). The salt stress had inhibitory effects on the tomato seedlings, including significant inhibitory effects on the chlorophyll content, transpiration rate, stomatal conductance, and Ci. Whether the decreased photosynthesis rate was related to both stomatal function (opening and closing) depended on the duration of salt stress, salt concentration, and crop species (**Figure 1**). The effect of stomatal and nonstomatal limitation mostly contributes to the changes of net photosynthetic rates in plants (Shaki et al., 2020; Navarro-León et al., 2021). However, whether the stomatal and nonstomatal limitation could decrease the photosynthetic rate of seedlings required further investigation.

When plants are exposed to salt stress, the content of enzymes and hormones would undergo dynamic changes and thereby initiate some physiological and biochemical processes which related to stress resistance to cope with environmental stress (Raghavendra et al., 2010; Yu et al., 2020). The stimulation of salt stress could rapidly increase the endogenous ABA levels and SnRK2s was activated by the enhanced ABA signaling (Umezawa et al., 2009; Kong and Ramonell, 2022). Besides, SnRK2s could phosphorylate the AREB/ABF, which could control stomatal closure in plants (Wen et al., 2022). Photosynthesis and transpiration were coupled through stomatal regulation and ABA played an important role in this process (Yu et al., 2020). A key step toward understanding the structure of the ABA signaling network for salt tolerance systematically was to obtain a comprehensive and accurate understanding of the dynamic transcriptional reprogramming and changes in ABA content that occurred in plants upon salt stress stimulation. We found that the endogenous ABA content in tomatoes increased rapidly after the salt treatment and *CYP707As* played an important role in promoting ABA biosynthesis. SnRK2-related genes were not differentially expressed, while *AAO3* and *NCED* were differentially expressed and phosphorylated ABF genes were significantly upregulated (**Figure 2**). This indicated that the progress in response to salt stress in tomatoes might mainly induce ABA accumulation by inducing the expression of *CYP707A* and *NCED* and then relay the signal to the *ABF*.

Previous studies demonstrated that SA could improve the salt tolerance of plants by increasing the photosynthesis efficiency (Jayakannan et al., 2015). In *Arabidopsis thaliana*, application of excessive SA (> 100 μM) could inhibite the seed germination, while exerting appropriate amount of SA (< 50 μM) could alleviate this inhibition (Lee et al., 2010). Overexpression of SA biosynthetic gene *MhNPR1* could increase salt tolerance of tobacco (Zhang et al., 2014). Overexpression of *AtNPR1* in rice could lead to salt sensitivity, which suggested that SA had a dose effect in regulating salt tolerance (Qiu et al., 2020). In tomatoes, the SA level increased significantly at 0.5 h under salt stress, suggesting that a larger amount of SA might be required for tomato salt tolerance (**Figure 3**). Previous studies of wheat and Arabidopsis thaliana had found that the enhancement of JA signal could exhibit enhanced salt tolerance (Zhao et al., 2014). In contrast, the enhancement of JA signal could exhibit greater salt tolerance in tomatoes (**Figure 3**) (Abouelsaad and Renault, 2018). GA could regulate growth throughout the plant life cycle (Zhou et al., 2020; UÇARLI, 2021). Therefore, according to changes in environmental conditions, the GA content was adjusted to regulate the relationship between stress and balance under salt stress conditions (Achard et al., 2006). Tomato might limit growth by reducing GA levels under salt stress. This suggested that JA and GA-mediated growth inhibition might be one of potential mechanisms for tomato salt tolerance.

The responses of single plant hormone to osmotic stress was investigated and ethylene and GA could mainly regulate the cell division and cell volume expansion, ABA could mediate the response of the mature plant cells to mild osmotic stress (Du et al., 2022). Some researchers speculated that the phytohormone signals might originate from other plant tissues rather than their own *de novo* synthetic pathways (Staswick, 2009). Additionally, this demonstrated that how hormone modifications were interrelated and likely affected one another. Tomatoes could establish new balance by dynamically regulating their hormone levels to adapt to salt stress (**Figures 2**, **4**).

In tomatoes, the number of identified transcription factors that were associated with abiotic stress resistance was relatively small (Du et al., 2017; Thirumalaikumar et al., 2018; Niu et al., 2022). Furthermore, the regulatory relationship between TFs involved in the abiotic stress resistance and their target genes had yet to be explored. WGCNA has been proved to be advantageous in detecting pairs of co-expression modules and hub genes (Deng et al., 2021). By analyzing the transcriptomic and hormonal profiling of root tissues in tomatoes, we established the co-expression network of hormones, TFs, and their target genes under salt stress for the first time (**Figure 7**). The synergistic relationships between hormone signals under salt stress was revealed for providing valuable insights into the molecular mechanisms of salt tolerance in tomatoes.

**Figure 8:**
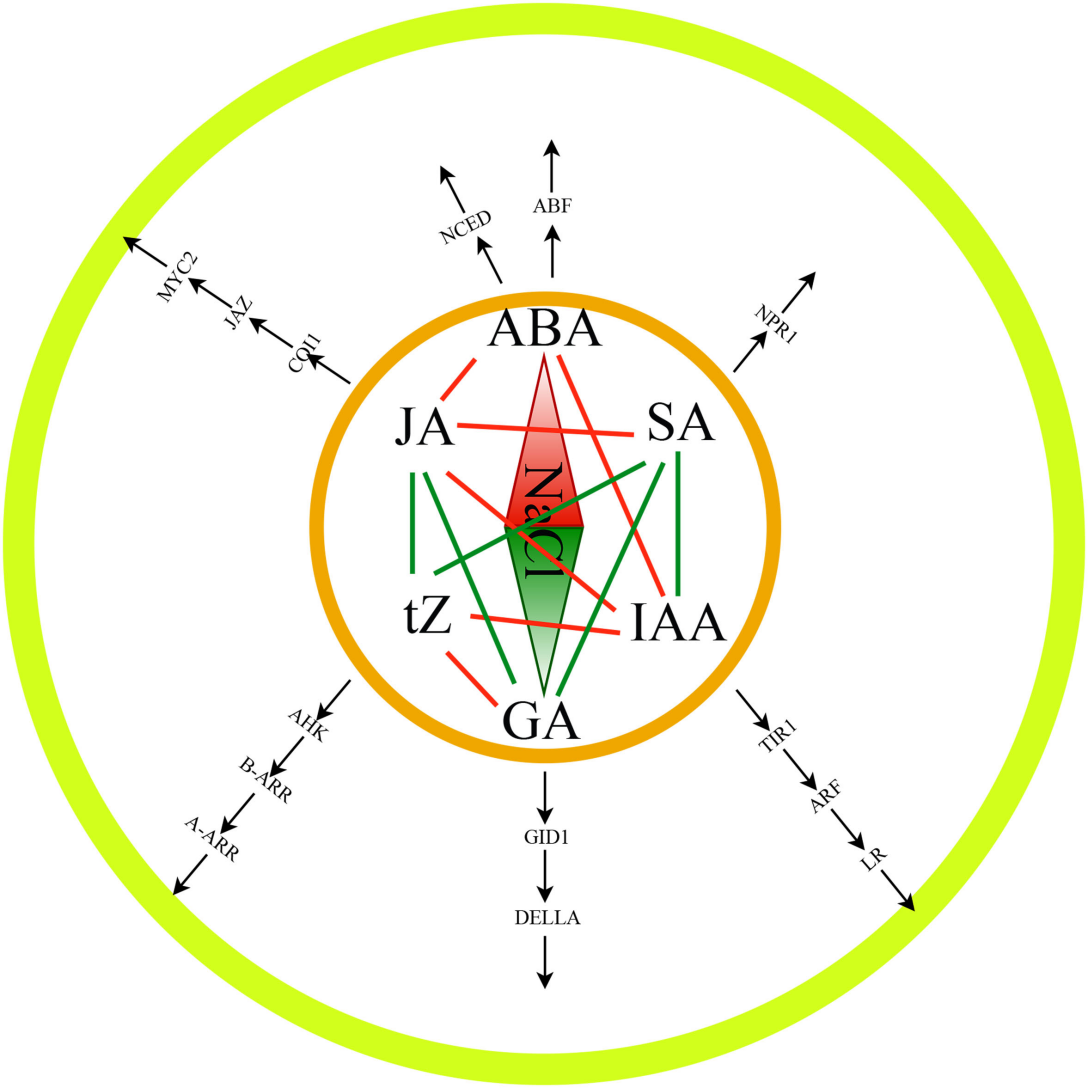
The plant hormone-mediated salt tolerance of tomato plants.

Finally, we performed the transcriptome and WGCNA analysis and identified several genes which involved in hormone synthesis and signal transmission by utilizing the RNA-Seq and hormone data from tomato root tissues. The links between hormones and their regulatory components, as well as the correlations between hormones, were examined (**Figure 8**). Additionally, the gene regulatory network of salt stress-induced tomato salt tolerance was constructed and a transcriptional regulation model of salt tolerance was also proposed. It contained 6 types of hormones, 14 types of TFs, and 52 target genes. This study not only provided a comprehensive overview of the interaction network of salt stress related hormones, TFs, and genes but also provided a basis for analyzing the molecular mechanisms of salt tolerance in tomatoes.

## 4 Materials and methods

### 4.1 Plant material and growing conditions

The cultivated tomato M82 was used as the experiment material for this study. Healthy and plump seeds were sown in 72-hole plug trays containing Nutrition Soil with vermiculite (soil: vermiculite, 2: 1). Similar development patterns among the seedlings were chosen, and they were then transplanted into a bucket with 12 L of 50% Hoagland solution. The plants were planted in a 12-liter bucket that had been treated with NaCl and completely filled with Hoagland solution. The photosynthetic indices were determined at 0 h, 0.5 h, 2 h, 6 h, 12 h, and 24 h after treatment, and the roots and leaves were sampled, weighed, and immediately frozen in liquid nitrogen at -80°C. Subsequently samples were utilized for physiology and biochemistry, hormone content, RNA-seq, and qRT-PCR testing and analysis.

### 4.2 Determination of ABA, JA, tZ, SA, IAA, and GA contents

The biological samples stored at -80°C were removed and ground with a grinder (30 Hz, 1 min) to a powder. Then, 50 mg of the ground samples were weighed, and 10 μL of the internal standard mixed solution with a concentration of 100 ng·mL^-1^ was added. Next, 1 mL of a 15: 4: 1 extractant (methanol, water, and formic acid) was added, mixed, and centrifuged for 5 minutes at 4°C and 12000 rpm. The tube was then reconstituted with 1 mL of an 80% methanol/water solution after concentration, put through a 0.22 m filter membrane, and placed in a sample vial. The tube was concentrated and used for liquid chromatography-tandem mass spectrometry (LC-MS/MS) to determine the amount of ABA, JA, tZ, SA, IAA, and GA.

### 4.3 Determination of physiological, biochemical, and photosynthetic indicators

The following settings were changed using a portable photosynthesis apparatus (CIRAS-3): CO_2_ was Ambient (remove chemixals), H_2_O was fixed of reference (100%), PAPi was 1000 umol m^-2^·s^-1^, Flow was Flow 300 (cc·min^-1^), leaf area was 4.5 (cm^2^), and the Pn, Tr, Ci, Gs, and WUE of the leaves were determined at 6 time points of salt stress treatment. Three biological duplicates of each sample were used to measure it. The cryopreserved biological samples were taken out using the Suzhou Keming Biotechnology Co., Ltd. kit’s procedure. For the physiological indicators: CAT, MDA, Pro, POD, PPO, SOD, and chlorophyll content, each indicator was determined in triplicate.

### 4.4 RNA-seq sequencing

The RNA-seq libraries of 18 samples were constructed using 3 biological replicates at 0 h, 0.5 h, 2 h, 6 h, 12 h, and 24 h after salt treatment, and the sequencing was performed on the Illumina HiSeq 2000 platform (Wuhan Metware Biotechnology Co., Ltd.). Data quality control and filtering were performed by fastp software (Chen et al., 2018), and the clean data were used for subsequent analysis. The tomato Heinz 1706 genome SL3.0 (https://solgenomics.net/ftp/tomato_genome/Heinz1706/) was used as the reference genome. Read alignment was carried out using HISAT2, and StringTie was used to assemble the aligned reads and estimate the transcripts abundance (Kim et al., 2015; Pertea et al., 2015).

### 4.5 Differential expression analysis

DESeq2 implemented in the R-project package was used to identify DEGs (Love et al., 2014). The threshold arguments of DEGs were set to padj  ≤  0.01 and FoldChange   ≥  2. The KEGG database (https://www.kegg.jp/ghostkoala/) was utilized for identifying hormone biosynthesis and signal transduction genes based on the DEGs (Kanehisa et al., 2021). All of the DEG protein sequences were uploaded to PlantTFDB (http://planttfdb.cbi.pku.edu.cn/) for TF analysis.

### 4.6 Construction of the co-expression network

The gene expression profiles of the DEGs were analyzed by the dynamic branch-cut method using the R package WGCNA (Langfelder and Horvath, 2008). To ensure the distribution of scale-free networks, the weighting coefficient β needs to satisfy the correlation coefficient close to 0.85 and have a certain degree of connectivity of each gene. In this study, β was set to 12. The automatic network construction function of block-wise modules was used to construct the network and multiple valid modules containing different number of genes were obtained. Min Module Size = 30 and Merge Cut Height = 0.25 were set as the standard, the modules with a similarity of 0.75 were merged. Specificity modules were screened with r > 0.80 and *P*-value < 0.05. Cytoscape (v3.9.1) software was used to visualize the co-expression networks (Doncheva et al., 2018).

### 4.7 qRT-PCR

Primer 6 software was utilized to design the phosphor quantification primers (**Table S1**) (Li and Brownley, 2010). Real-time PCR amplification was performed on a Roche LightCycler 96, and the reactions for each sample were carried out in triplicate. The ChamQ Universal SYBR qPCR Master Mix (Vazyme, China) kit was used to create a 20 L amplification apparatus according to kit instructions. Each cycle consisted of pre-denaturation at 94°C for 30 s, denaturation at 94°C for 5 s, annealing at 60°C for 5 s, and extension at 72°C for 34 s. A total of 40 cycles were run. The results were analyzed for relative quantification using the 2^–ΔΔCt^ method.

## Data availability statement

The original contributions presented in the study are publicly available. This data can be found here: NCBI, PRJNA888477.

## Author contributions

Conceptualization, QY and NL Methodology, BW and JW Software, TY Validation, JXW, QD and FZ Formal analysis, FZ and RX Investigation, QD and RX Resource collection, Data curation, JXW and RX. Writing-original draft preparation, BW and JW. Writing-review and editing, QY and NL. Visualization, BW. Supervision, QY and NL. Project administration, NL. Funding acquisition, QY and NL. All authors contributed to the article and approved the submitted version.
