# Supplementary figures and images for: The transcriptional regulatory network of hormones and genes under salt stress in tomato plants (Solanum lycopersicum L.)
Source: Front Plant Sci. 2023 Feb 6;14:1115593. doi: 10.3389/fpls.2023.1115593 (PMC9939653; doi:10.3389/fpls.2023.1115593)

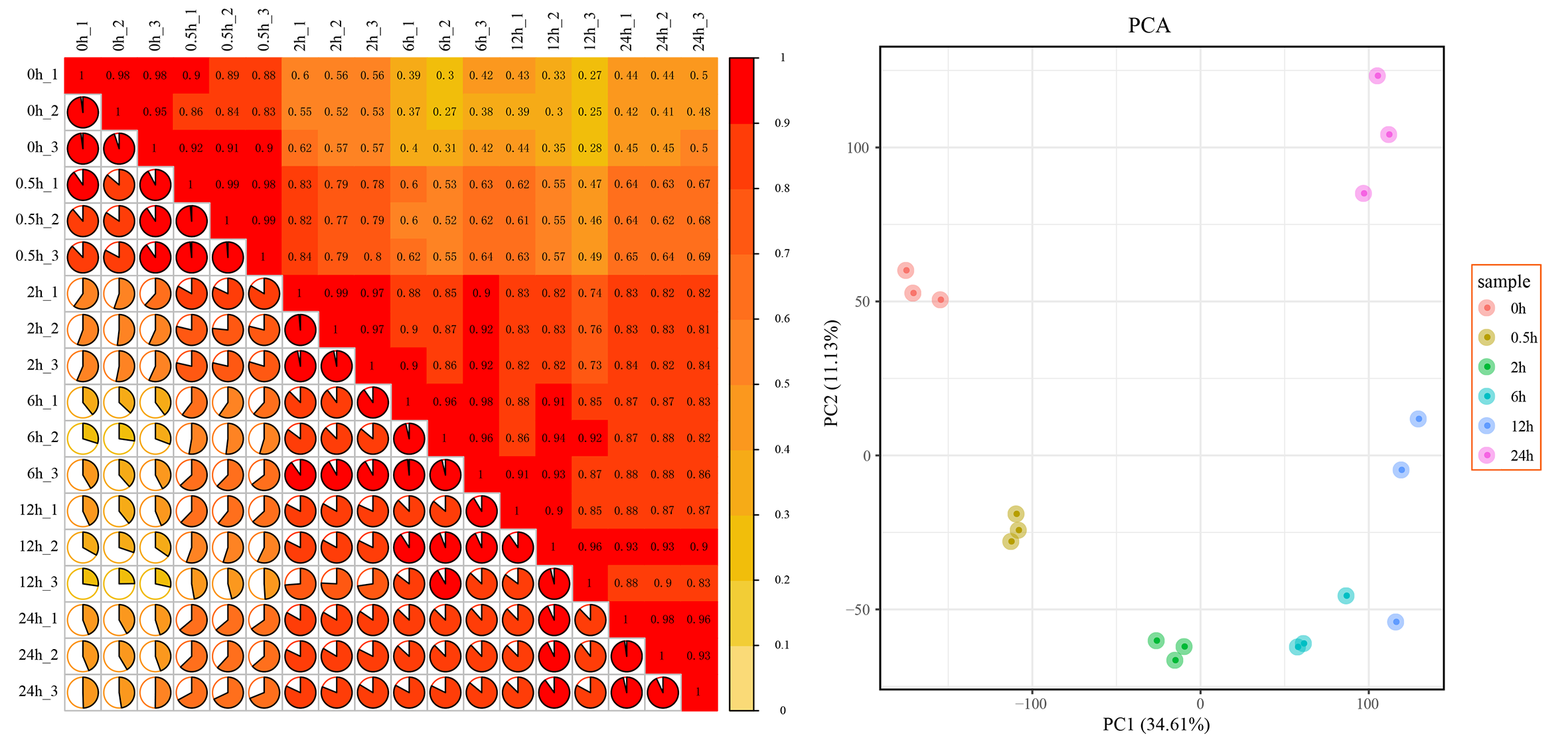

Supplement: Supplementary file 1 [file Image_1.tif]

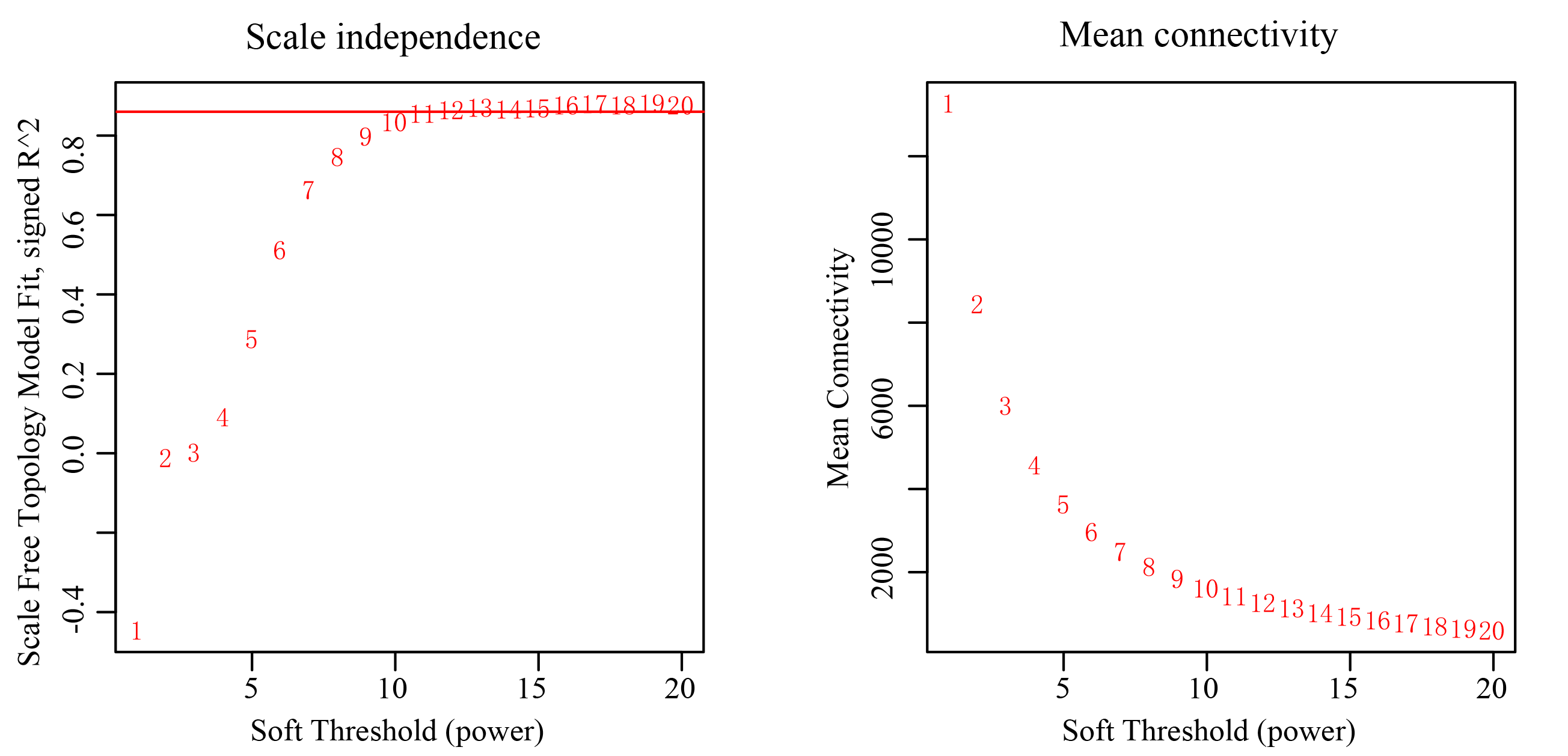

Supplement: Supplementary file 2 [file Image_2.tif]

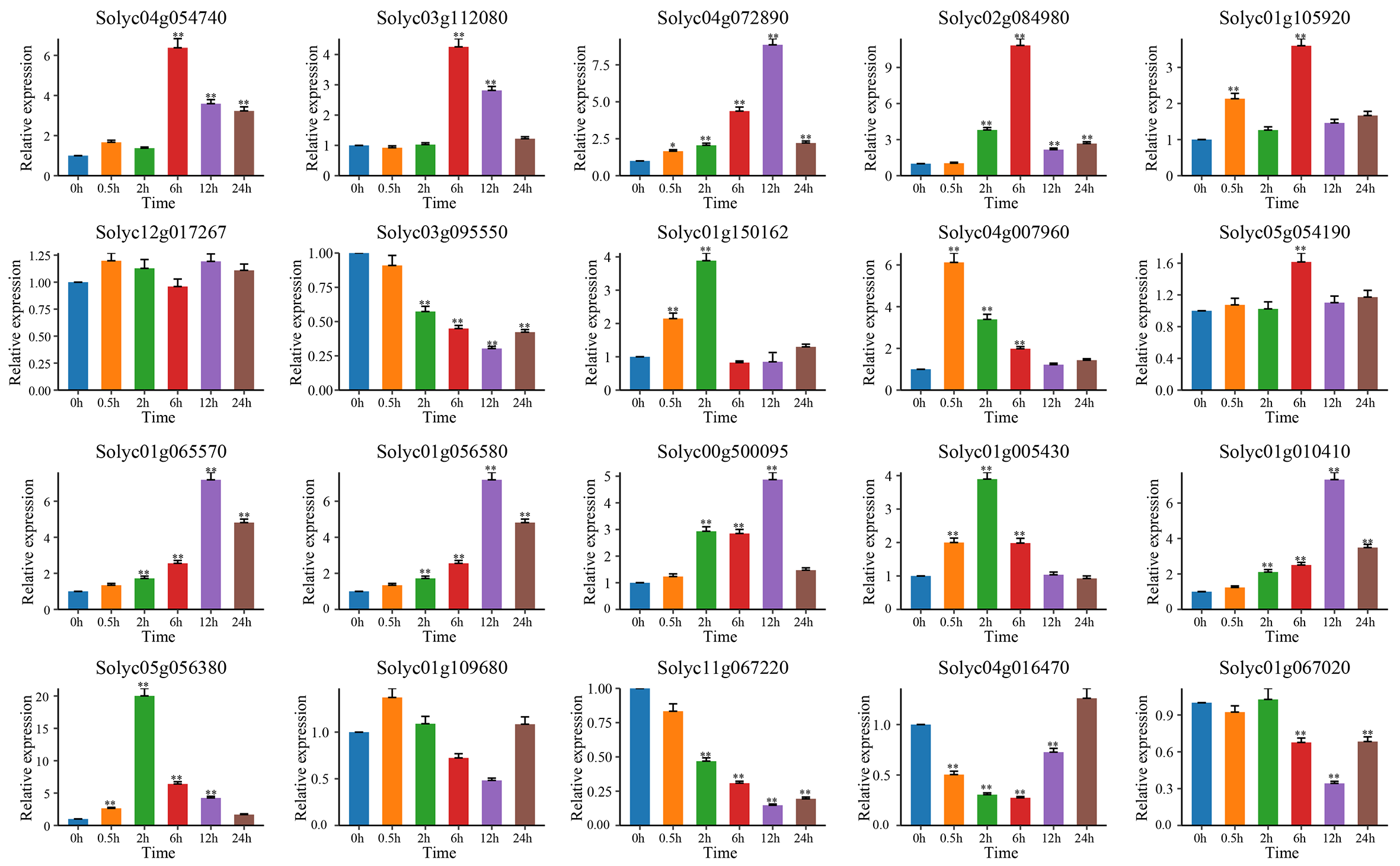

Supplement: Supplementary file 3 [file Image_3.tif]

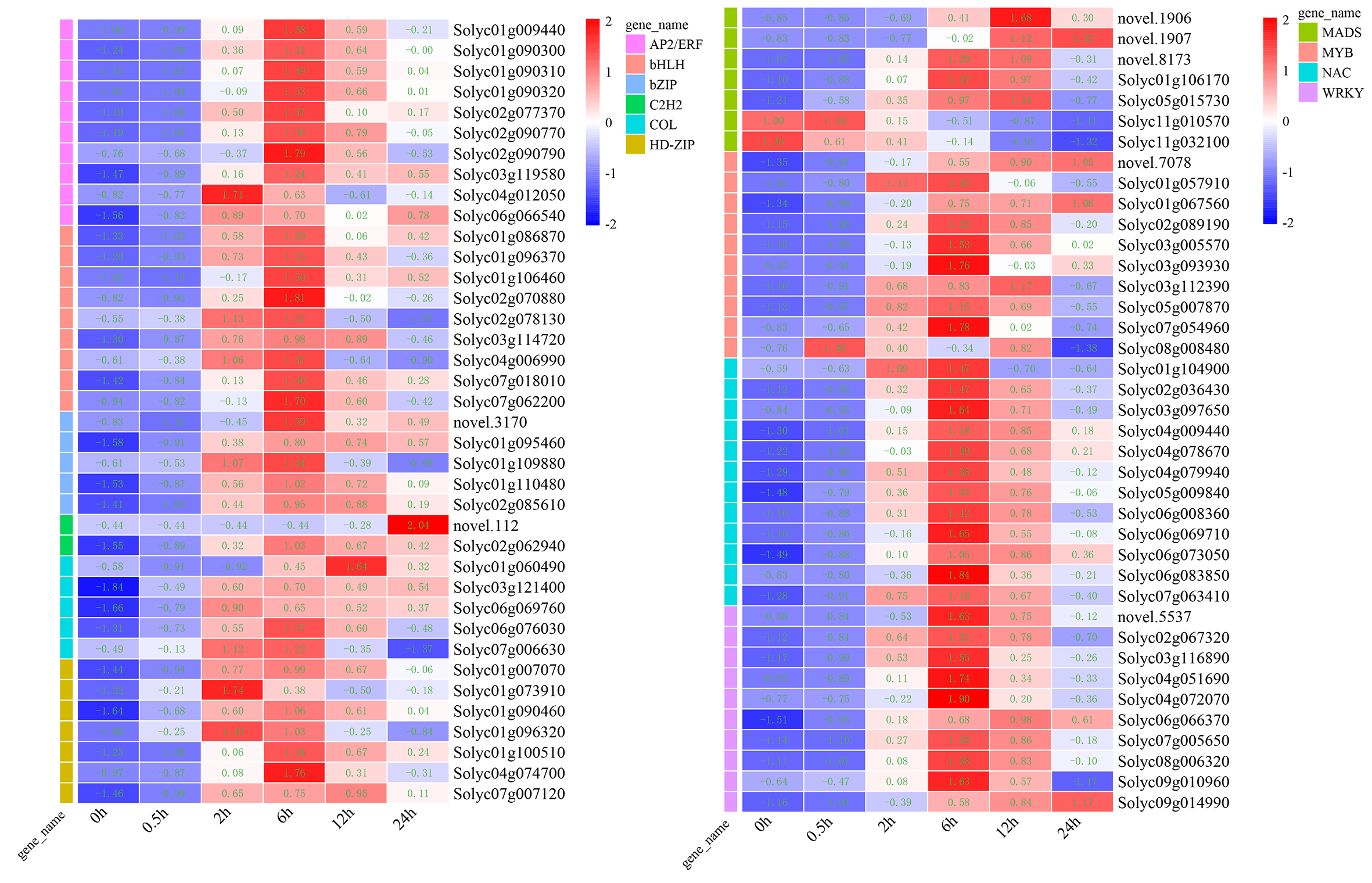

Supplement: Supplementary file 4 [file Image_4.tif]

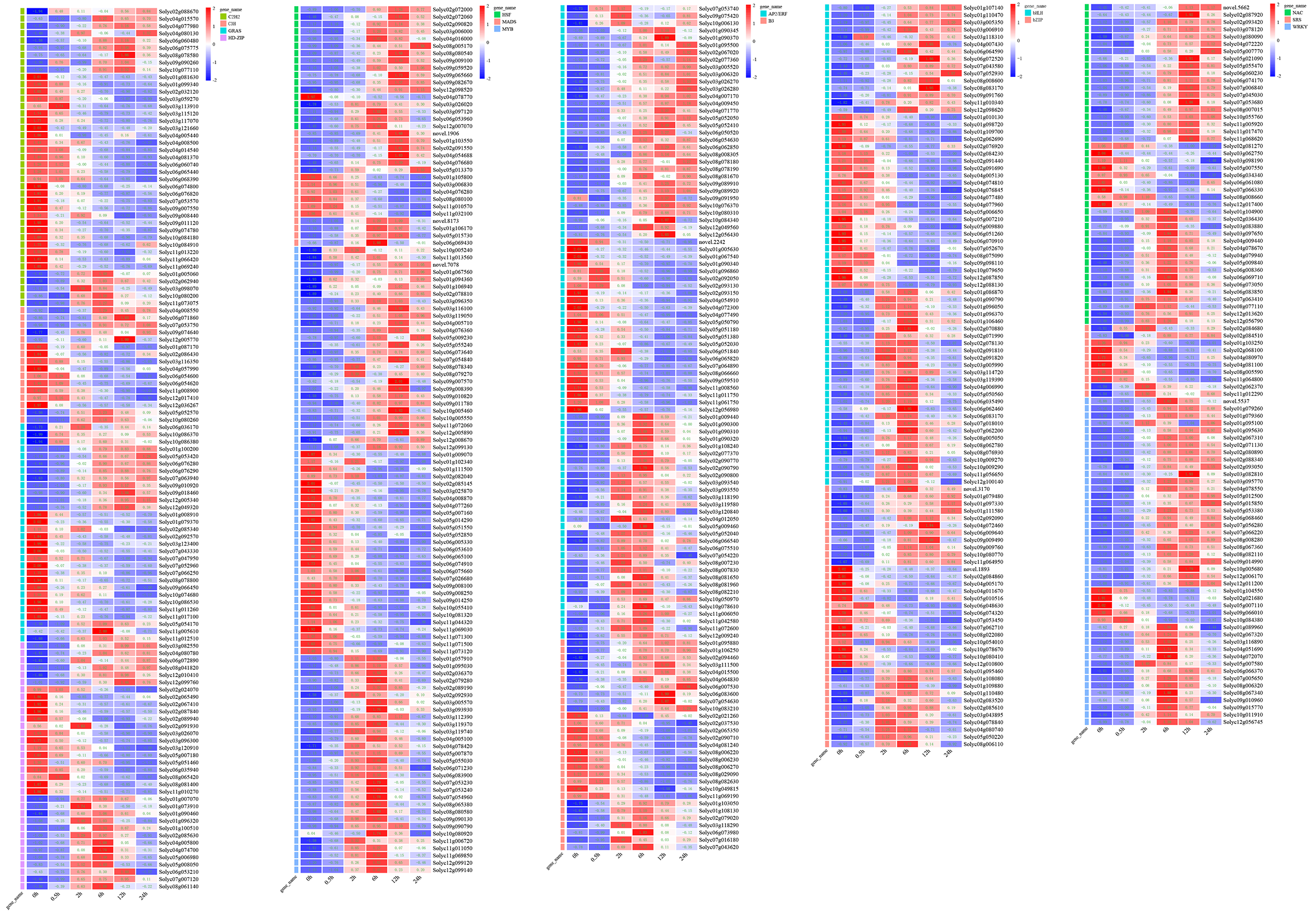

Supplement: Supplementary file 5 [file Image_5.tif]
